# A Survey of Multifingered Robotic Manipulation: Biological Results, Structural Evolvements, and Learning Methods

**DOI:** 10.3389/fnbot.2022.843267

**Published:** 2022-04-27

**Authors:** Yinlin Li, Peng Wang, Rui Li, Mo Tao, Zhiyong Liu, Hong Qiao

**Affiliations:** ^1^State Key Laboratory for Management and Control of Complex Systems, Institute of Automation, Chinese Academy of Sciences, Beijing, China; ^2^School of Artificial Intelligence, University of Chinese Academy of Sciences, Beijing, China; ^3^Centre for Artificial Intelligence and Robotics, Hong Kong Institute of Science and Innovation, Chinese Academy of Sciences, Hong Kong, China; ^4^School of Automation, Chongqing University, Chongqing, China; ^5^Science and Technology on Thermal Energy and Power Laboratory, Wuhan Second Ship Design and Research Institute, Wuhan, China; ^6^School of Automation Science and Electrical Engineering, Beihang University, Beijing, China

**Keywords:** multifingered hand, visual-motor control, multi-mode fusion, hand structural evolution, learning-based manipulation

## Abstract

Multifingered robotic hands (usually referred to as dexterous hands) are designed to achieve human-level or human-like manipulations for robots or as prostheses for the disabled. The research dates back 30 years ago, yet, there remain great challenges to effectively design and control them due to their high dimensionality of configuration, frequently switched interaction modes, and various task generalization requirements. This article aims to give a brief overview of multifingered robotic manipulation from three aspects: a) the biological results, b) the structural evolvements, and c) the learning methods, and discuss potential future directions. First, we investigate the structure and principle of hand-centered visual sensing, tactile sensing, and motor control and related behavioral results. Then, we review several typical multifingered dexterous hands from task scenarios, actuation mechanisms, and in-hand sensors points. Third, we report the recent progress of various learning-based multifingered manipulation methods, including but not limited to reinforcement learning, imitation learning, and other sub-class methods. The article concludes with open issues and our thoughts on future directions.

## 1. Introduction

Robotic grippers have been successfully applied in the manufacturing industry for many years, showing an elevated level of precision and efficiency and enjoying a low cost. However, how to make a robotic hand with the same dexterity and compliance as the human hand has attracted much attention in recent years due to the urgent demand for robot applications in our daily life and many unstructured complex environments, such as service robots for disabled people and rescue robots for disasters, in which robotic or prosthetic hands need to interact with various targets.

There are still many challenges for the design, control, and application of multifingered robotic dexterous hands. From the design aspect, designing a multifingered robotic hand in a limited space, as well as integrating multimodal distributed sensors and high precision actuators are difficult, not to mention satisfying the weight and payload requirements at the same time. From the learning and control aspect, due to the high dimensionality of states and action spaces, frequently switched interaction modes between multifingered hands and objects, making the direct using of typical analytic methods and learning from scratch methods for two-fingered grippers arduous. Moreover, from the application aspect, the multifingered robotic hand and its algorithms are always personalized, which will limit their transfer and adaptation for widespread applications, as the "internal adaptation" for the body, software, and perception variations, and "external adaptation" for the environment, object, and task variations are intractable (Cui and Trinkle, 2021). Along with the related studies, benchmark experimental environments and tasks to fairly and comprehensively compare and evaluate the various properties, such as precision, efficiency, robustness, safety, success rate, adaptation, are urgently needed.

Due to the development of neuroscience and information science as well as new materials and sensors, a series of robotic hands and their learning and control methods are designed and proposed. Among them, on the one hand, mimicking the perception, sensor-motor control and development structures, mechanisms and materials of the human hand is a promising way, such as flexible and stretchable skins, multimodal fusion, and synergy control (Gerratt et al., 2014; Ficuciello et al., 2019; Su et al., 2021). On the other hand, deep learning based representation learning, adaptive control concerning uncertainties, learning-based manipulation methods, such as deep convolutional neural network (CNN), reinforcement learning, imitation learning, and meta-learning (Rajeswaran et al., 2017; Yu et al., 2018; Li et al., 2019a; Nagabandi et al., 2019; Su et al., 2020), show significant superiority for robotic movement and manipulation learning and adaptation.

In this article, focusing on multifingered dexterous hands (no less than three fingers are considered), the typical and novel work in neuroscience and robotics from three aspects: a) the biological results, b) the structural evolution, and c) the learning methods are reviewed. By reviewing these correlative studies, especially their cross-over studies, we hope to give some insights into the design, learning, and control of multifingered dexterous hands. Note that there are some reviews concerning the structure, sensors of robotic hands, and control and learning for robotic grasping, assembly, and manipulation (Bicchi, 2000; Yousef et al., 2011; Mattar, 2013; Controzzi et al., 2014; Ozawa and Tahara, 2017; Bing et al., 2018; Billard and Kragic, 2019; Kroemer et al., 2019; Li and Qiao, 2019; Mohammed et al., 2020; Cui and Trinkle, 2021; Qiao et al., 2021), while none of them unfold from these three aspects for multifingered dexterous hands with no less than three fingers (refer to Table 1).

**Table 1:** Comparisons of the existing reviews.

**Literature**	**Perspective**
Bicchi (2000)	• reviews robotic hand designs in terms of human operability, manipulation dexterity and grasp robustness
Yousef et al. (2011)	• presents the SOTA tactile sensing techniques for robotic hands • reveals the characteristics of the human hands during in-hand manipulation
Mattar (2013)	• presents the SOTA on biomimetic based dexterous hands • indicates the significance of bio-inspired thinking in robotic hand design
Controzzi et al. (2014)	• presents the SOTA robotic hand designs in terms of prosthetics and humanoid robotics • focuses on the human hand function and its inspiration to the design of robotic hands
Ozawa and Tahara (2017)	• reviews the grasping and dexterous manipulation studies from the perspective of control
Li and Qiao (2019)	• reviews the works on high-precision robotic manipulation from the aspects of sensing-based/compliant-based/environmental constraint-based/sensing-constraint hybrid/others
Qiao et al. (2021)	• reviews the brain-inspired models for robots in vision, decision, motion control and musculoskeletal systems
Kroemer et al. (2019)	• describes a formalization of the robot manipulation learning problem with a single coherent framework
Mohammed et al. (2020)	• reviews the deep reinforcement learning based object grasping methods
Bing et al. (2018)	• surveys the bio-inspired spiking neural networks for robotic control task
Billard and Kragic (2019)	• describes the trends and challenges in robot manipulation
Cui and Trinkle (2021)	• summarizes the types of variations for robot manipulation and categorizes and contrasts learned robot manipulation methods with adaptation
This work	• reviews the biological results of perception and motor of hand manipulation • reports the SOTA designs of dexterous hands and reviews the learning-based manipulation studies focusing on multifingered robotic hands

**Figure 1:**
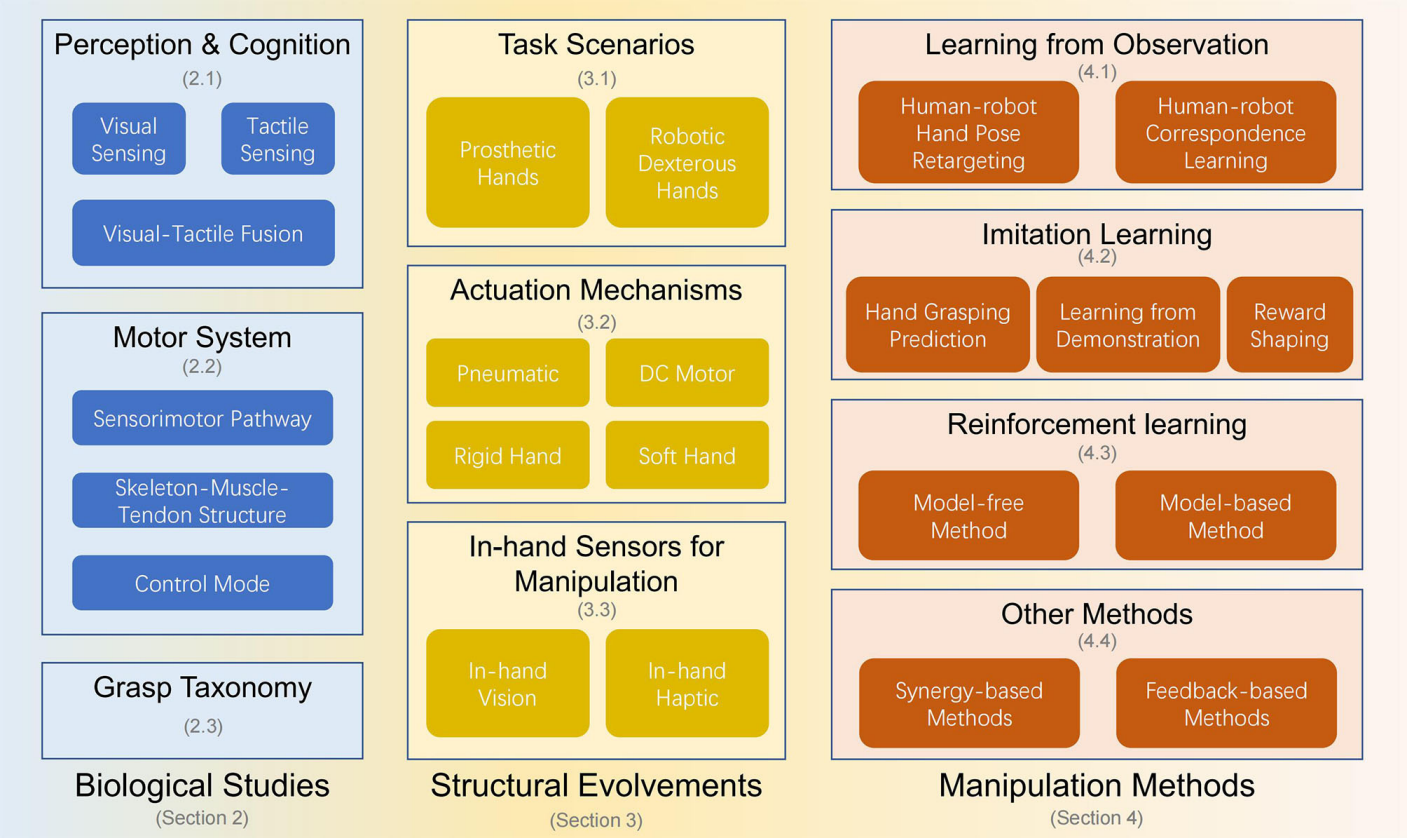
The brief overview of the organization of the article.

A brief overview of the organization of this article is given in Figure 1. First, the anatomical, physiological and ethological studies of the hand of the primate, mainly humans, are investigated (Section 2), including the pipeline, structure, and function of multimodal perception and cognition, motor control, and grasp taxonomy. Second, the structural evolvements of hand are surveyed from three perspectives: the task scenario, the actuation mechanism, and the in-hand sensors (Section 3). Third, by distinguishing the availability and type of supervision data, three types of learning-based grasp and manipulation methods, learning from observation (LfO), imitation learning, and reinforcement learning are reviewed, and two sub-classes containing the synergy-based methods and feedback-based methods are also enrolled (Section 4). Finally, open issues are discussed (Section 5). We believe that human-like multifingered robotic hand design, control and manipulation learning methods could promote the interdisciplinary development of robotics and neuroscience.

## 2. Biological Studies

The hand is the most important interaction component of humans. Even for normal hand manipulation in daily life, various perception, cognition and motor control brain regions, sensor units, and physical organs are necessarily required and effectively cooperated. In this section, we review some anatomical, physiological and ethological studies related to hand manipulation, hoping to provide some insights into the algorithm research and the hardware design of multifingered hands in the future.

### 2.1. Perception and Cognition

The human hand perceives environmental information from the surroundings *via* the following three aspects: a) mechanoreceptors and thermoreceptors in the skin, b) proprioceptive inputs from the muscles and joints, and c) centrally originating signals. For manipulation, the perception of hands is frequently combined with vision. Specifically, visual sense can provide the class, shape, position, pose, velocity, affordance properties, etc. information of the target from task planning (reaching process) until manipulation ends, while tactile sense can provide real-time and dynamic contact and force feedback during the grasping and non-prehensile manipulation process, which is the key for compliant, robust and dexterous manipulation. Tactile sensors become more important for unknown objects and dark environments, as general knowledge and visual sense may be partially unavailable.

#### 2.1.1. Visual Sensing

**Figure 2:**
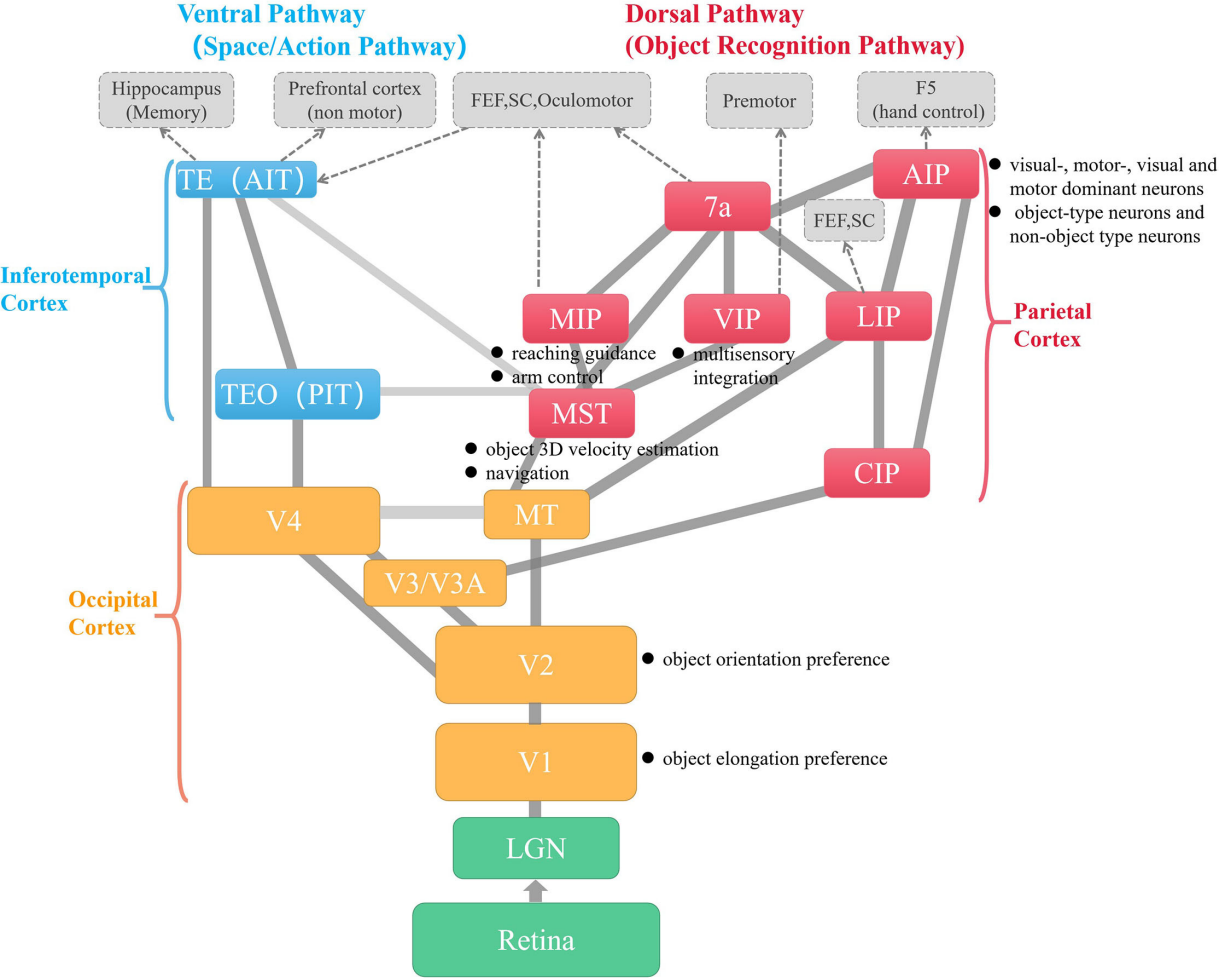
The visual and dorsal pathways of the primate visual cortex and their connected regions. Excepting retina and LGN in green, the yellow, red, and blue blocks are areas that are in the occipital cortex, parietal cortex, and inferotemporal cortex, respectively. The gray blocks with dotted borders are areas in other cortexes related to motor perception, learning, and control. The black texts with dot marks following each block explain the area's functions from the manipulation aspect (Goldman-Rakic and Rakic, 1991; Prevosto et al., 2009; Kruger et al., 2013).

**Visual pathways**: As shown in Figure 2, the primate visual cortex has two pathways, the ventral pathway and the dorsal pathway. In common knowledge, the ventral pathway is responsible for object recognition and is simplified as *what* pathway; the dorsal pathway is responsible for object localization and is simplified as *where* pathway. Particularly for hand manipulation, the dorsal pathway is also found to provide visual guidance for the activities of human-object interaction, such as reaching and grasping (Culham et al., 2003). For complex object-centered hand movements, such as skilled grasp, the interaction between the two pathways could enhance their functions iteratively. It is hypothesized that the dorsal pathway needs to retrieve the detailed identity properties saved in the ventral pathway for fine-tuning grasps, while the ventral pathway may need to obtain the latest grasp-related states from the dorsal pathway to refine the object internal representation (van Polanen and Davare, 2015).

**Hand-centered visual modulation**: A series of neurophysiological and ethological studies suggest that visual processing near the hand is altered, mainly reflected in visual manipulation property selection and enhancement in accuracy and effectiveness, such as preferred orientation selection of objects in V2 (Perry et al., 2015), object elongation preference in V1 and pSPOC, selection of wrist orientation and visual extraction of object affordances in aSPOC, and object shape and number of digits encoding in aIPS (Fabbri et al., 2016; Perry et al., 2016).

Neurons in the AIP area have been classified as “visual-dominant”, “motor-dominant”, and “visual and motor” classes (Taira et al., 1990). Moreover, there are two kinds of visual dominant neurons: object-type neurons and non-object type neurons. Biologists suggest that the former represents the shape, size, and/or orientation of 3D objects, and the latter represents the shape of the handgrip, grip size, or hand orientation (Murata et al., 2000). Meanwhile, V6A, as a part of the dorsolateral stream, has a function similar to AIP. Their difference may be the division of work, as AIP seems to be responsible for both object recognition and visual monitoring of grasping, while V6A seems to be mainly involved in the visual guidance of reach-to-grasp. Another possibility is that AIP is more involved in slow, finer control and V6A is more involved in fast, broad control (Breveglieri et al., 2016).

#### 2.1.2. Tactile Sensing

Human hands are sophisticated yet sensitive sensory systems, which are more complicated than arms, owing to their anatomical structure and nerve supply (Bensmaia and Tillery, 2014). Tactile sensing, as part of the haptic (touch) feedback sensing system of human hands, is achieved relying on skin stimulation.

**Structure of tactile sensor distribution**: Years of research has recognized the anatomic structure of the hand skin. The skin consists of three layers: the epidermis, the dermis, and the subcutaneous layer, from the outer layer to the inner layer.

There are four types of sensory receptors underneath the different layers of the skin of the human hands: slowly adapting type 1 (SA1, Merkel Cells), slowly adapting type 2 (SA2, Ruffini Corpuscle), rapidly adapting type 1 (RA1, Meissner's Corpuscle), and rapidly adapting type 2 (RA2, Pancinian Corpuscle). Each of the types corresponds to different aspects of skin deformation (Johnson, 2001). For instance, the SA1 unit has small receptive fields (RFs) and produces a tonic response to steady skin deformation. This type is sensitive to pressure. The RA1 unit also has small RFs but produces a phasic response to skin deformation. It is rapidly adapting and sensitive to flutters. The RA2 unit has large RFs and is sensitive to high-frequency vibrations. The SA2 unit is sensitive to sustained skin deformation. Both SA1 and SA2 produce a continuous response. The four types of sensory receptors function differently for manipulation (Dahiya et al., 2010). For instance, the SA1 unit is heavily used in texture perception. The SA2 unit is used for skip detection. The RA1 unit is used in motion detection. The RA2 unit is highly involved in tool use processes.

The fiber plexuses, which transmit either motor or sensory neural electric signals, are embedded within the subcutaneous layer. These fibers form a large, complicated network that connects sensory receptors (or free nerve endings) to the spinal cord or the central nervous system. Correspondingly, there are a vast number of mechanoreceptors connected to the fiber plexuses. A report shows that there are up to 500 mechanoreceptors per cubic centimeter of the human hand on average, thus achieving a very sensitive touch reaction, with a resolution for displacements of the skin as small as 10 nm (Jones and Lederman, 2006; Pruszynski and Johansson, 2014).

**Pathway, function, and mechanism**: The sensory receptors (or mechanoreceptors) and fiber plexuses in the skin transmit signals to the spinal cord or brainstem *via* receptor neuron cells. There are three major components of the cells (Nicholls et al., 2012): a) A neuronal cell located in the dorsal root ganglion of the spinal conus foramen. b) A central branch that converges to the dorsal root and projects to the spinal cord. c) A peripheral branch that converges with other nerve fibers to form a peripheral nerve and terminates to form a specialized receptor complex. These peripheral axons are usually long, and they generate action potentials remarkably close to the nerve terminal and travel past the ganglion cell cytosol into the spinal cord or brainstem.

Observation of the activity of tactile fibers by micro-nerve recording reveals that most nerve fibers have little or no spontaneous activity and discharge only when the skin is stimulated. When the human hand touches an object, the cells within the sensory receptors are squeezed, and the layers formed by the cells are displaced, producing neural electric signals to transmit through the fiber plexus to the spinal cord or the central nervous system.

In addition, for hand manipulation, tactile RFs in the somatosensory cortex are smaller than their counterparts in M1 and proprioceptive areas. As cutaneous sensors encode local shape features, including curvature and edge orientation, at the contact point (Bensmaia et al., 2008; Yau et al., 2013), smaller RFs could represent elaborate tactile spatial information, while the RFs of neurons in M1 and proprioceptive areas include several joints spanning the entire hand (Saleh et al., 2012; Goodman et al., 2019).

#### 2.1.3. Visual-Tactile Fusion

Neurons activated by visual, tactile, and samatosensory stimuli are found in multiple brain regions of the primate, such as the VIP and premotor cortex (PMC) (Graziano and Gandhi, 2000; Avillac et al., 2005), and multisensory inputs are integrated into linear (additive) or nonlinear (non-additive) manners (Gentile et al., 2011). Non-additive integration could increase the overall perception ability and movement performance both in efficiency and sensitivity, with which weak and threshold bellowed signals could be detected and the saliency of one modality could be enhanced by another modality with attention modulation. Meanwhile, enhancement is realized when the signals are congruent in both spatial and temporal proximity for the target event, which is explained as cross-modal stimuli falling with the unified RFs of the same neurons (Stein and Stanford, 2008). If they are not synchronized, the activation of neurons may even be lower than the single stimulus situation (der Burg et al., 2009).

Specifically, for the hand reaching and grasping process, multisensory (visual, tactile, and somatosensory) perceptions are dynamically modulated, as tactile suppression is stronger for hand reaching to facilitate the processing of somatosensory signals and then weaker for fingers that are involved in the grasping process (Gertz et al., 2018). In addition, visual hand images could implicitly enhance visual and tactile integration, making them hard to distinguish in the temporal domain (Ide and Hidaka, 2013). Except for dominant modal switching when other modalities are not available, some researchers try to find the multisensory integration mode of the redundant positional and size cues and suggest that haptic position cues, not haptic size, are integrated with visual cues to achieve faster movements with smaller grip apertures, which could then better scale visual size (Camponogara and Volcic, 2020). Moreover, some researchers investigate the difference between the right and left hands, which indicates that the right hand prefers visually guided grasping for fine motor movement, while the left hand specializes in haptically guided object recognition and spatial arrangement (Morange-Majoux, 2011; Stone and Gonzalez, 2015). From ethology and statistics aspects, it is also found that by simply observing hand exploration motion, humans or algorithms can coarsely estimate the tactile properties of objects (Yokosaka et al., 2018).

### 2.2. Motor System

Motor control of mammals is organized in a hierarchical mode, and various studies on motivation, planning, and motor pattern generation have been explored. In this section, we first briefly introduce the motor pathways and the sub-areas related to hand motor planning and control. Then, the detailed skeleton-muscle-tendon structure of the hand and its control modes, such as population coding and multi-joint coordination for reaching and grasping, are investigated.

#### 2.2.1. Sensorimotor Pathway

**Figure 3:**
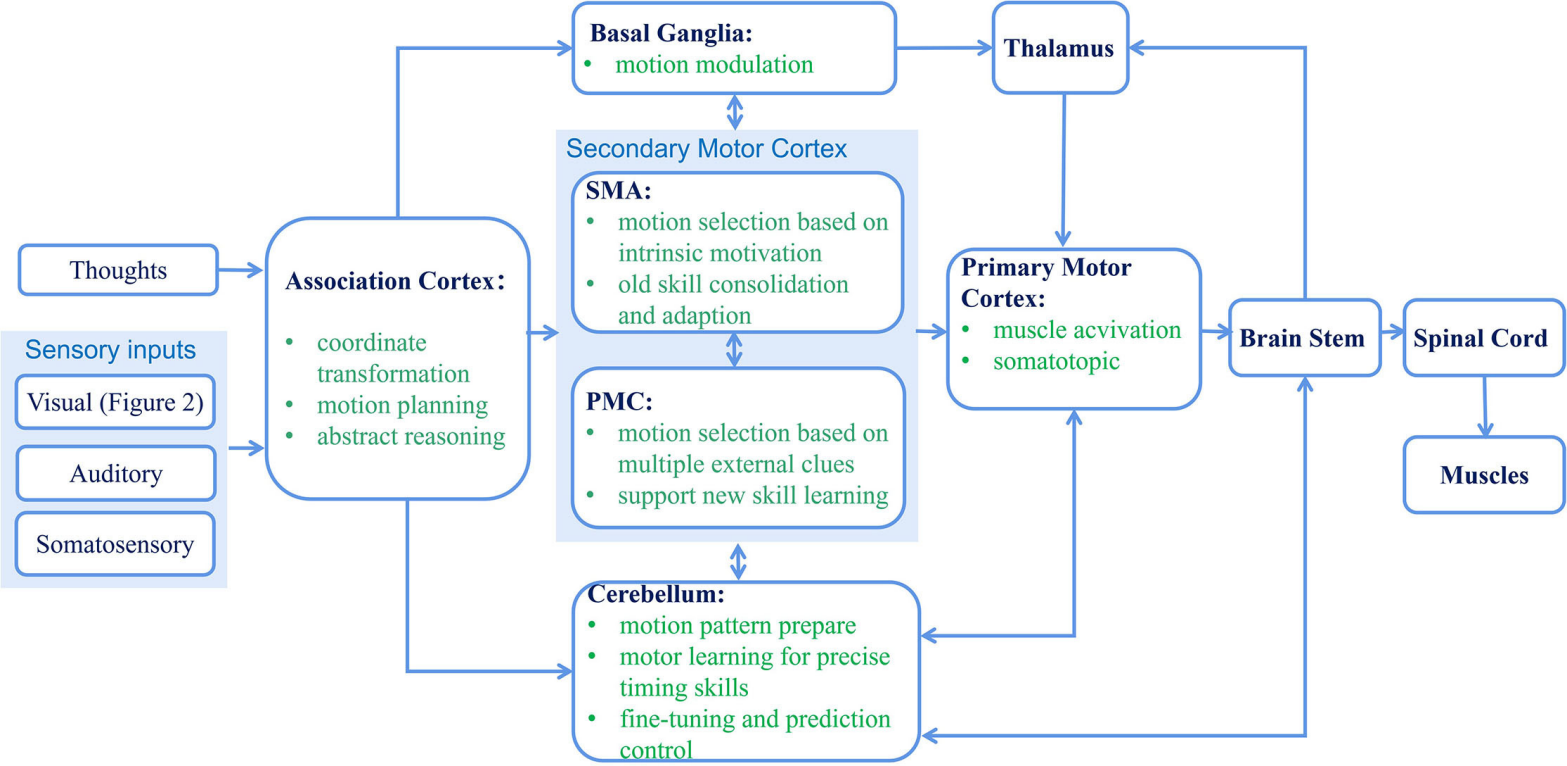
Simplified diagram of the sensorimotor system. The green texts in each block explain the functions of the corresponding area. Note that various cross-layer and inter-layer feedforward and feedback connections existed for sensorimotor modulation and adaptation, which are not shown in this figure for brevity (Middleton, 2000; Shmuelof and Krakauer, 2011; Gazzaniga, 2014).

A simplified diagram of the sensorimotor system is shown in Figure 3. The association cortex mainly includes the posterior parietal cortex (PPC) and dorsolateral prefrontal cortex (DLPFC) and is involved in body part coordinate transformation, motor planning, abstract reasoning, etc. (Kroger, 2002; Buneo and Andersen, 2006; Kuang et al., 2015). Particularly, PPC integrates various sensory information and acts as a sensorimotor interface for motion planning and online control of visual guided arm-hand movements (Buneo and Andersen, 2006). Then, there are two discrete circuits for further motor planning. The external loop contains the PMC, cerebellum, and parietal cortex. As this loop connects with multiple sensor regions, it may be guided by external clues and support new skill learning. The internal loop includes the supplementary motor area (SMA), prefrontal cortex, and basal ganglia, which may be modulated by intrinsic motivation and prefer old skill consolidation and adaptation. Finally, all their outputs are sent to the motor cortex for further muscle activation (Middleton, 2000; Gazzaniga, 2014). In addition, the cerebellum could provide a feedforward sensory prediction allowing for prediction control, and the basal ganglia reinforce better action selection, which is necessary for the early acquisition of novel sequential actions (Shmuelof and Krakauer, 2011).

More specifically, in the homunculus map of the motor cortex and somatosensory cortex, the hand acts as an effector that projects to a large cortex region despite its small size on the human body scale. This reflects its importance and prominent level in control and sensing. Various areas in the premotor are also involved in object-grasping control. For example, F5 receives signals from both motor- and visual-dominant neurons in the AIT area and realizes visual-motor transformation (Michaels and Scherberger, 2018); sub-area F5a could extract 3D features of objects and plan cue-dependent grasp activity. Area F6 controls *when* and *how* (grip type) to grasp (Gerbella et al., 2017).

#### 2.2.2. Skeleton-Muscle-Tendon Structure

**Skeleton**: The human arm is made up of three bones - the upper arm bone (humerus) and two forearm bones (the ulna and the radius), forming 6 joints (the sternoclavicular, acromioclavicular, shoulder, elbow, radioulnar, and wrist joints) and 7 degrees-of-freedom (DoFs) of movement. As a result, the human hand has a far more complicated bone structure - i.e., 27 bones, forming 15 joints and 21 DoFs of movement, including the wrist (Jones and Lederman, 2006).

It is worth mentioning that the skeletal structure of the metacarpophalangeal (MCP) joint preserves excellent features that allow the fingers to execute both adduction/abduction and flexion/extension motions, thus, increasing the dexterity of the overall system (Nanayakkara et al., 2017). Kontoudis et al. (2019) designed a tendon-driven modular hand inspired by the MCP to perform concurrent flexion/extension and adduction/abduction with two actuators.

**Musculotendon units**: The muscles of the upper limb form an overly complex system and can be divided into three classes: a) muscles of the shoulder, b) muscles of the humerus that act on the forearm, and c) muscles of the wrist and hand. Many of the muscles are further recognized as extrinsic and intrinsic musculotendon units. The extrinsic muscles, which contain long flexors and extensors, are located on the forearm to control crude movement; while intrinsic muscles are located within the hand and are in charge of fine motor functions (Li et al., 2015).

For each hand, 38 extrinsic and intrinsic musculotendon units are attached to the bones that control the movement of the hands (Tubiana, 1981). One end of these extrinsic hand muscles is fixed at the bones in the arm, and the muscles stretch all along to the wrist and become tendons at the wrist. The intrinsic muscles appear within the hands and are divided into four groups: the thenar, hypothenar, interossei, and lumbrical muscles (Dawson-Amoah and Varacallo, 2021). There are numerous robotic systems that attempt to mimic the tendon structure of human hands and arms, from the structure to the power source. Some of the successful systems include the Anatomically Correct Testbed (ACT) hand (Rombokas et al., 2012), Myorobotics (Jantsch et al., 2012, 2014), and Roboy (Martius et al., 2016; Richter et al., 2016).

#### 2.2.3. Control Mode

Hand reach-grasp manipulation is a continuous control process composed of five phases. First, the hand is transported by the arm, and the arm moves in a bell-shaped velocity (Fan et al., 2005). Second, hand pre-shapes to adapt to the target object, which occurs in the last 30-40% of the reaching process (Hu et al., 2005). Third, grasp is determined by form closure, force closure, functional affordance, grasp stability, grasp security, etc. (Scano et al., 2018). Fourth, the manipulation phase includes hand-arm coordinated move and/or switched interaction modes between object and hand. Finally, the release of the object and return of the hand and arm are all categorized into five phases. During the process, proprioceptive and M1 neurons show a strong preference for time-varying hand postures during grasping, in contrast to their intense preference for time-varying velocities during reaching (Goodman et al., 2019).

Population vector is a popular motion coding mode that could indicate the orientation of motion and has a successful application in the brain-machine interface (BMI). For hand manipulation task, acting as a motor pattern generator, M1 exhibit low-dimensional population-level linear dynamics during reach and reach-to-grasp (Churchland et al., 2012; Rouse and Schieber, 2018). However, for the grasp task, the dynamics seem higher-dimensional or more nonlinear. One reason may be that grasp is primarily driven by more afferent inputs rather than intrinsic dynamics (Suresh et al., 2020) (as shown in Section 2.1). The other reason could be that the activity of single neurons in the motor cortex relates to the movements of more than one finger (Valyi-Nagy et al., 1999), and one neuron can drive the facilitation and suppression of several muscles (Hudson et al., 2017). Thus, we could draw a conclusion that muscles of the hand are controlled in a level of multi-joint coordination pattern. Compared to the independent control method, this control manner is a slightly higher abstraction and could benefit generation, learning of new skills, and simplify planning (Merel et al., 2019). Multi-joint coordination is also consistent with the control mode found in the central nervous system (CNS), which is commonly named muscle synergies (Taborri et al., 2018), it is defined as the coherent activation of a group of muscles in space and time. For hand grasp and manipulation, both spatial and temporal muscle synergies are analyzed in the primate Overduin et al. (2008); Jarrassé et al. (2014), by applying decomposition method, such as PCA, control could be realized in the low dimensional subspace of hand configuration, as 2–4 synergies could account for 80–95% variance of hand motions.

The population vector is a popular motion coding mode that can indicate the orientation of motion and has a successful application in brain-machine interfaces (BMIs). For the hand manipulation task, acting as a motor pattern generator, M1 exhibits low-dimensional population-level linear dynamics during reach and reach-to-grasp (Churchland et al., 2012; Rouse and Schieber, 2018). However, for the grasp task, the dynamics seem higher-dimensional or more nonlinear. One reason may be that grasp is primarily driven by more afferent inputs rather than intrinsic dynamics (Suresh et al., 2020) (as shown in Section 2.1). The other reason could be that the activity of a single neuron in the motor cortex relates to the movements of more than one finger (Valyi-Nagy et al., 1999), and one neuron can drive the facilitation and suppression of several muscles (Hudson et al., 2017). Thus, we could conclude that muscles of the hand are controlled at a level of multi-joint coordination. Compared to the independent control method, this control method has slightly higher abstraction and could benefit the generation and learning of new skills as well as simplify planning (Merel et al., 2019). Multi-joint coordination is also consistent with the control mode found in the central nervous system (CNS), which is commonly named muscle synergies (Taborri et al., 2018); it is defined as the coherent activation of a group of muscles in space and time. For hand grasp and manipulation, both spatial and temporal muscle synergies are analyzed in the primate Overduin et al. (2008). By applying decomposition methods, such as PCA, control could be realized in the low-dimensional subspace of hand configuration, as 2–4 synergies could account for 80–95% variance of hand motions (Jarrassé et al., 2014).

In addition, concerning the hierarchical structure of motor control, except complex connections and feedback between different regions in Figure 3, which could be useful for best and adaptive motion modulation, the regions also have some kind of autonomy and amortized control ability. This is especially true for low-level controllers and effectors, which can quickly generate simple, repetitive, reusable motions without sensor and planning inputs from high levels (Merel et al., 2019). This is evidenced by experiments in which monkeys can walk, climb, and pick up objects when their bilateral dorsal root ganglions (DRGs) are damaged, and a patient without proprioception ability can shape different gestures in a dark environment (Rothwell et al., 1982).

### 2.3. Grasp Taxonomy

Except for the aforementioned results and hypotheses, ethological studies on grasp taxonomy concerning hand kinematics, constraints of each grasp, and common use patterns are meaningful for hand activity recognition, rehabilitation, biomechanics, etc. (Feix et al., 2016; Gupta et al., 2016b).

By considering different grasp attributes, a series of studies on grasp taxonomy are proposed. The pioneering study of Schlesinger et al. divides human grasps into six categories—cylindrical, tip, hook, palmar, spherical, and lateral—based on object properties (Borchardt et al., 1919). Then, Niper's landmark work on power and precision grips takes the intention of activity as an import control principle (Napier, 1956). For manufacturing applications, Cutkosky builds a grasp taxonomy in a hierarchical manner by taking the constraints of tasks, hands, and objects into consideration, and an expert system and grasp quality measure derived from analytical models are developed (Cutkosky, 1989). Recently, by investigating a) the opposition type, b) the virtual finger assignments, c) the type in terms of power, precision, or intermediate grasp, and d) the position of the thumb, Feix et al. (2016) divide hand grasps into 33 types, which could be further reduced to 17 classes if the object shape/size is ruled out. In addition to ethological studies, the effectiveness of grasp attributes for fine-grained hand manipulation activity recognition has also been proven in information science. A simple encoding of known grasp type, opposition type, object, grasp dimension, and motion constraints integrated with multi-class classifiers has achieved an accuracy of 84% for 455 activity classes (Gupta et al., 2016b).

## 3. Structural Evolvements

With nearly 40 years of development, much research on the structural design of dexterous hands has been conducted. Some of them have become well-known products, such as Shadow Hand (Reichel, 2004; Kochan, 2005), BarrettHand (Townsend, 2000), DLR-Hand (Butterfass et al., 2001), and RightHand^1^.

There have been several reviews about dexterous hands in recent years (Bicchi, 2000; Yousef et al., 2011; Mattar, 2013; Controzzi et al., 2014; Ozawa and Tahara, 2017). Most of the literature discusses the research in this area from the aspects of a) kinematic architecture, b) actuation principles, c) actuation transmission, d) sensors, e) materials, and f) manufacturing methods. To avoid redundancy and focus on dexterous manipulation, this survey will re-organize the works from three perspectives: a) the task scenario, b) the actuation mechanism, and c) the sensors for manipulation.

### 3.1. Task Scenarios

**Figure 4:**
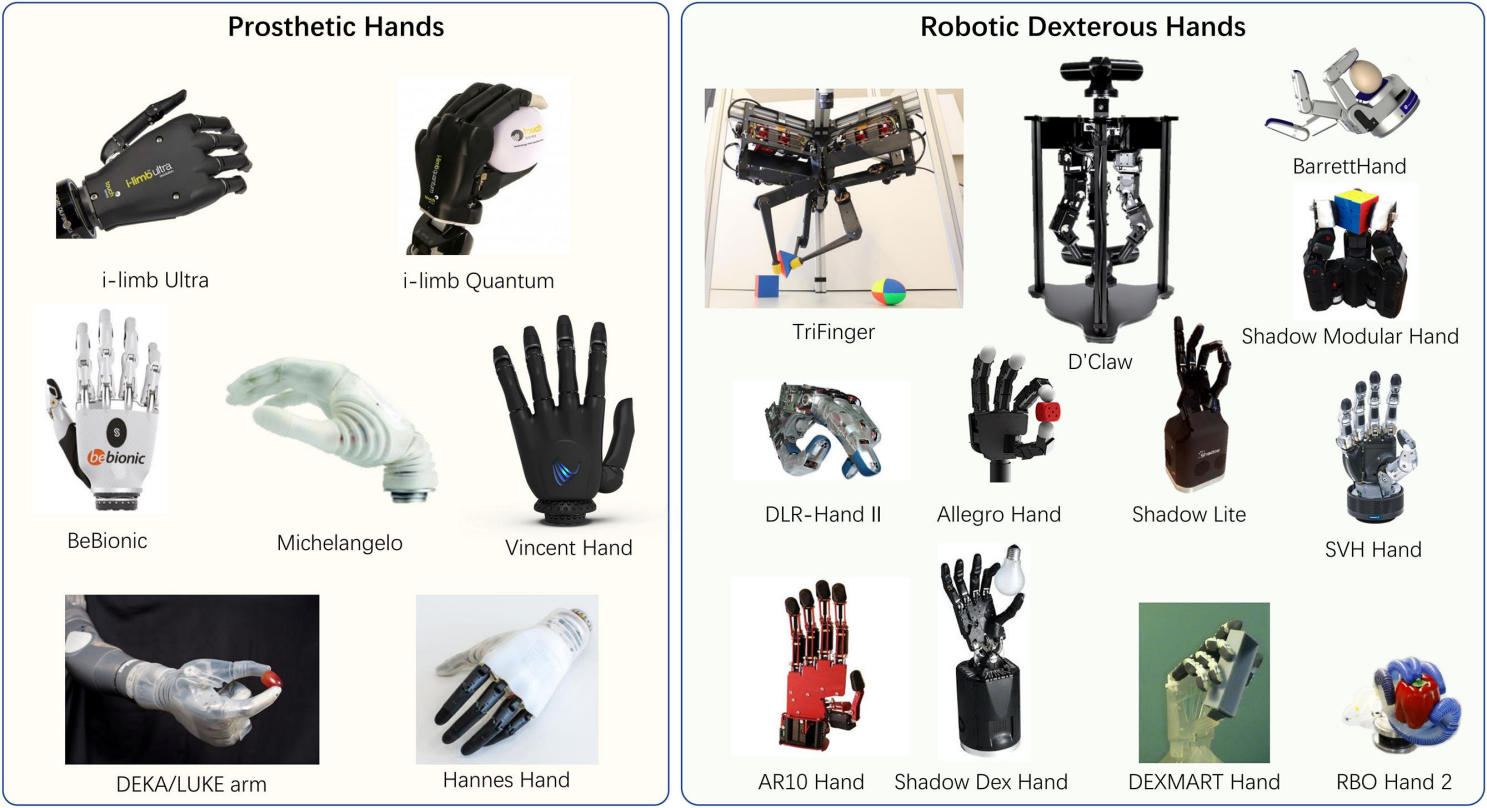
Some of the current designs of dexterous hands.

Dexterous hands are typically used as part of the prosthetic hand for the disabled or as the end-effector of a manipulator for robots (refer to Figure 4). For the former scenario, the goal is to design a dexterous hand that is as close to a human hand as possible. Most of the designs choose a five-fingered structure. Since the last two decades, there have been a series of prosthetic hand products - i-limb Quantum/Ultra by Touch Bionics^2^, BeBionic^3^ and Michelangelo^4^ by Ottobock, and the Vincent hand^5^ from Vincent systems. Meanwhile, new designs and products continue to appear, such as the DEKA/LUKE arm developed by DEKA Integrated Solutions Corp. for US crew members with upper limb loss from Iraq or Afghanistan (Resnik et al., 2014), and the Hannes hand that mimics a series of key biological properties of the human hand (Laffranchi et al., 2020).

For the latter scenario, there are massive variants of designs, ranging from simplified three-fingered hands to five-fingered hands, due to their specific applications, such as a) three-fingered: TriFinger (Wuthrich et al., 2020), D'Claw (Ahn et al., 2020), Shadow Modular Grasper (Pestell et al., 2019), BarrettHand (Townsend, 2000); b) four-fingered: DLR-Hand II (Butterfass et al., 2001), Allegro hand (Veiga et al., 2020), Shadow Dexterous Hand Lite; c) five-fingered: AR10 Robotic Hand (Devine et al., 2016), A Gesture Based Anthropomorphic Robotic Hand (Tian et al., 2021), The DEXMART hand (Palli et al., 2014), Shadow Dexterous Hand (Reichel, 2004; Kochan, 2005), RBO Hand 2 (Deimel and Brock, 2013). Tables 2, 3 listed some of the technical details of the robotic hands mentioned in this article.

**Table 2:** The technical details of the prosthetic hands.

**Name**	**No. fingers/grip patterns**	**Gripping force/Payload**	**Weight**	**Time from full open to full close**
i-limb Ultra	5/18	Hand load limit: 40−90kg Finger load limit: 20−32kg	0.432−0.528 kg	0.8 s
i-Limb Quantum	5/36	Hand load limit: 40−90 kg Finger load limit: 20−48 kg	0.432−0.558 kg	0.8 s
BeBionic	5/14	Hand load limit: 40 kg Finger load limit: 25 kg	0.402−0.689 kg	0.5−1.0 s
Michelangelo	5/7	Gripping force: 70 N/ 60 N/ 15 N (Opposition Mode/Lateral Mode/Natural Mode)	0.52 kg	N/A
VINCENTevolution4	5/15	Hand load limit: 35 kg Finger load limit: 12 kg	0.39−0.56 kg	0.6 s
DEKA/LUKE arm	5/6	N/A	1.4 kg	N/A
Hannes Hand	5/−	Gripping force 150 N	0.45 kg	1 s

**Table 3:** The technical details of the robotic dexterous hands.

**Literature**	**Name**	**No. fingers/joints/DoFs**	**Dimensions**	**Weight**	**Payload**	**Year of appearance**	**Comment**
Wuthrich et al. (2020)	TriFinger	3/9/9	350 ×350 ×600 mm	N/A	N/A	2021	An open-source robotic platform intended to support research in dexterous manipulation
Ahn et al. (2019)	D'Claw	3/9/9	127 ×127 ×226 mm	N/A	N/A	2019	A platform for exploring learning-based techniques in dexterous manipulation
Townsend (2000)	BarrettHand	3/9/4	335 ×89 ×102 mm	0.98 kg	6.0 kg	2000	The long-established, flexible robotic hand
Pestell et al. (2019)	Shadow Modular Grasper	3/9/9	210 ×210 ×244 mm	2.7 kg	2.0 kg	2019	The modular design for industrial and research applications
Butterfass et al. (2001)	DLR-Hand II	4/13/13	150 ×150 ×300 mm	1.8 kg	30 N	2001	The fully actuated multi-sensory hand for space robot
/	Shadow Dexterous Hand Lite	4/16/13	135 ×135 ×448 mm	2.4 kg	Up to 4.0 kg	2015	A streamlined version of the shadow dexterous hand
/	Allegro hand	4/16/16	65 ×135 ×239 mm	1.09 kg	Up to 5.0 kg	2016	Lightweight and portable anthropomorphic design robotic hand
Jacobsen et al. (1986)	Utah/MIT Hand	4/16/38	Comparable to a human hand but with a huge cable driver	N/A	N/A	1986	The first cable-driven robotic hand
Bridgwater et al. (2012)	Robonaut 2 Hand	5/14/14	127 ×127 ×304 mm	N/A	More than 9 kg	2011	The fully actuated dexterous hand for space manipulation
Ruehl et al. (2014)	SVH Hand	5/20/9	92 ×90 ×242 mm	1.3 kg	N/A	2014	The first robot gripper approved by the German Social Accident Insurance (DGUV) for collaborative operation
/	AR10 Humanoid Robot Hand	5/10/10	Comparable to a human hand	N/A	N/A	2016	A standard servo actuated humanoid hand design
Kochan (2005)	Shadow Dexterous Hand	5/24/20	135 ×135 ×448 mm	4.3 kg	Up to 4.0 kg	2005	An anthropomorphic design robotic hand that is comparable to the human hand in terms of size and structure
Palli et al. (2014)	The DEXMART hand	5/20/24	Comparable to a human hand but with a big cable driver	N/A	2.7 kg	2014	A recreated design in reference of human hand as design and behavioral model
Deimel and Brock (2013)	RBO Hand 2	5/∞/7	80 ×80 ×130 mm	0.178 kg	Up to 0.5 kg	2013	A soft, pneumatic, compliant robotic hand

With the improvement of electronics and computational resources, the designs of dexterous hand systems for prosthetics and robotic manipulation are gradually evolving from less to more DoFs, rigidity to flexibility, and no sensing to multi-sensing fusion. In particular, distinctive design concepts have been derived in terms of their sizes, input signals, etc.

The sizes of the dexterous hands vary in different scenarios. The prosthetic hands are undoubtedly designed to be of the same size as a human hand; the robot hands, however, differ in size due to their research goals. For example, D'Claw (Ahn et al., 2020) is proposed as a benchmark platform to evaluate learning-based dexterous manipulation algorithms. The size of the “fingers” is in fact too large to be treated as “fingers”. However, such a design reduces the cost of motors and mechanics while maintaining dexterity in DoFs.

The input signals for robotic hands also vary in different scenarios. The prosthetic hands usually involve the acquisition of bioelectrical signals (EMG, electromyography, or the myoelectric signal), enabling the mapping of the human EMG to the joint variables of the fingers or the grasping patterns of the hand (Furui et al., 2019). Furthermore, to make the disabled grasp better, much work has been done in recent years to close the above “EMG-control” open-loop system by providing electric or vibration stimuli to the arm (Tyler, 2016; George et al., 2019). Such closed-loop systems guarantee the disabled to perform force-sensitive tasks such as grasping a blueberry without breaking. The robot hands, however, get their control values directly from the defined tasks. The control values are calculated in real time from the sensor input. In recent years, to improve the generalization of dexterous manipulation, these control values have often been extracted through a deep neural network with inputs of visual information (Fang et al., 2020).

### 3.2. Actuation Mechanisms

Unlike humans, muscle-like actuators do not exist for robotic hands. Due to the differences in the power source, the dexterous hands can be classified into pneumatic-powered hands and electric-powered hands. The pneumatic-powered hands appear earlier, but the large, noisy pumps made them inapplicable for non-factory scenarios (Jacobsen et al., 1985). In recent studies, many soft-fingered robotic hands have been actuated by pneumatic devices (Hubbard et al., 2021). On the other hand, due to advances in electric engineering, DC motors make it possible to build a robotic hand that is comparable to a human hand in size.

In terms of the number of actuators, the dexterous hands can be divided into two categories: a) fully-actuated, if the number of actuators equals the number of joints, and b) under-actuated, if the number of actuators is less than the number of joints (which means some of the joints are controlled by a shared actuator). Many early prototypes adopted under-actuated design since a fully-actuated dexterous hand usually has more than 20 actuators (Kochan, 2005), which brings great difficulty in finding a suitable controller. Meanwhile, evidence also shows that human hands are under-actuated. With the recent development of computer science, some neural networks have been built to solve the controller problem of fully-actuated hands (Andrychowicz et al., 2019).

The joints of the dexterous hands are actuated in two ways: a) each joint is actuated by an actuator at the position of the joint, and b) each joint is actuated by an actuator, which transmits its power from elsewhere. The former are usually seen by the fully-actuated hands, while the latter are under-actuated. Similar to human hands, which use tendons to transmit power from the muscles, many under-actuated hands use nylon or steel strings to simulate tendons and muscles and, therefore, create tendon-driven dexterous hands.

### 3.3. In-hand Sensors for Manipulation

The human hand has thermoreceptors, mechanoreceptors, and nociceptors, which ensure that the person can manipulate objects within a comfortable range and that he or she does not suffer serious injury (Nicholls et al., 2012). Unlike the human hand, the sensors deployed on the palm or finger surface of the dexterous hand are designed to be more task-oriented, ensuring that the task can be completed primarily. For this purpose, the in-hand sensors of the dexterous hand consist of visual sensors and haptic sensors. Vision sensors are used to obtain local visual information during operation - this sensor is mostly used in autonomous dexterity tasks; haptic sensors are used to obtain information about the contact force between the robot and the object during operation and can be further divided into tactile sensors and force sensors. The tactile sensors are more inclined to the details of the fingertips and palms of the hand, and the force sensors, on the other hand, are laid out at the finger joint locations and are used to sense more macroscopic force information.

**In-hand vision**: In-hand vision is a setup in which vision sensors are integrated into the robot hand and move with it. Eye-in-hand is not a human-like or human-inspired representation. However, due to the lack of flexibility of robot vision compared to that of humans, eye-in-hand is widely used in vision-related robotic system setups to help robots obtain local visual information between objects in contact with them during operation, thus enabling a variety of fine-tuned manipulation-oriented adjustments to be made.

In-hand vision is often combined with to-hand vision (or referred to as eye-to-hand) to obtain more comprehensive visual features for robotic manipulation (Flandin et al., 2000). With advances in depth vision sensors, such cameras (e.g., Kinect, RealSense) have become miniaturized and can be more easily integrated into the end-effector of a manipulator. Using such in-hand sensors, object-oriented 3D surface models can be constructed, thus providing better results for more dexterous grasping of 3D objects.

**In-hand haptic**: For humans, haptic feedback is divided into two different classes: tactile and kinesthetic. The former refers to the sense one feels in his/her fingertips or on the surface. The related tissue has many different sensors embedded in and underneath the skin. They allow the human brain to feel things such as vibration, pressure, touch, and texture. The latter refers to the sense one feels from sensors in his/her muscles, joints, tendons, such as weight, stretch, or joint angles of the arm, hand, wrist, and fingers.

For the dexterous hand, researchers have put earnest effort into realizing such a sensitive tactile sensor as a human fingertip. A variety of physics and material science principles have been applied to design tactile sensors of varying sizes, measuring ranges and sensitivities, forming resistive sensors, capacitive sensors, piezoelectric sensors, and optical sensors (Yousef et al., 2011).

In recent years, vision-based tactile sensors have drawn massive attention due to their easy development and economic maintenance (Shah et al., 2021). Such sensors use cameras to recognize the textures, features, or markers on a sensing skin and then compare their difference in task space with (or without) an illumination system to reconstruct the skin deformation, so as to acquire contact force information on the surface.

## 4. Learning-Based Manipulation Methods

From the manipulation methods aspect, there are analytic methods and data-driven methods/learning-based methods. The analytic methods typically analyze the physical characteristics of objects to achieve dexterity, stability, equilibrium, and dynamic behaviors (Shimoga, 1996; Kleeberger et al., 2020). However, it is difficult to obtain complete modeling of objects, thus could not adapt to complicated and volatile environments. While learning-based methods have attracted much attention in robotic manipulation as well as computer games and autonomous vehicles due to their high data efficiency, empirically evaluation manner, and good generation ability (Kroemer et al., 2019; Vinyals et al., 2019; Kiran et al., 2021). However, compared to common arm-gripper manipulation and autonomous vehicle, learning based manipulation of multifingered hand has new challenges: a) higher dimensionality of states and action spaces, such as the dimensionality of action vector is 30 for UR10 with Shadowhand; b) more complex and various tasks and environments, such as frequently switched interaction modes for in-hand manipulation task; c) more difficulties in direct kinematics teaching and cross-hardware/internal adaptation due to the big differences in structure, actuator, and DOF of each multifingered hand, etc, which make direct application of the common learning-based methods difficult.

In this section, by distinguishing the availability and type of supervision data, we try to classify and review the current studies in learning-based multifingered robotic hand manipulation methods for various tasks. In particular, only robotic dexterous hands with no less than three fingers are investigated. Note that classification according to various tasks, such as grasping, non-prehension manipulation, in hand manipulation, handover, etc could also be meaningful, as different task requirements influence the design of learning-based methods, which will not be specially discussed in this article.

### 4.1. Learning From Observation

Due to the high-dimensional state and action spaces of multifingered robotic hands, learning from human hand demonstration is a direct method. There are four crucial issues in this situation: a) 6-DoF object pose detection, b) human hand skeleton/pose/grasp type recognition, c) interaction mode perception between object and hand, and d) human-robot hand pose retargeting and manipulation learning. Various robotic systems and algorithms have been proposed to record demonstrations properly and solve issues partly or fully.

For LfO, we mainly consider the situation in which demonstrations are conducted by different subjects or from different aspects, in which action labels are not available and the state values are also barely known. Hand manipulation prediction and learning from demonstration with the same robotic/human hand will be reviewed in Section 4.2.

#### 4.1.1. Human-Robot Hand Pose Retargeting

For the four-fingered Allegro hand with 20 joint angles, DexPilot (Handa et al., 2020) defines and obtains 20 joint angles of the human hand in a multistage pipeline, which includes pre-perception of the hand mask and pose with a color glove, human pose detection and refinement based on PointNet++, and joint angle mapping based on an MLP net. However, due to the substantial difference in joint axes and positions, a cost function for kinematic retargeting concerning fingertip task-space metrics is proposed. Finally, complex vision-based teleoperation tasks such as extracting money from a wallet and opening a tea drawer are realized in a real robotic system. Taking human hand depth images as inputs, Antotsiou et al. (2019) use a hand pose estimator (HPE) to achieve hand skeleton extraction and then combine inverse kinematics and the hybrid PSO method to retarget to a hand model with 23 actuators, which is taken as ground truth for further Generative Adversarial Imitation Learning (GAIL). In contrast, Garcia-Hernando et al. (2020) realize retargeting in a similar way, which is taken as noisy mapping requiring further correction. Different from the multistage methods, Li et al. (2019a) adopt a BioIK solver to construct a dataset with 400K pairwise depth images of the human hand, the Shadow Hand, and its joint angles first. Then, a teacher-student network with joint angle, consistency, and physical loss is trained to directly give angle joints of the Shadow Hand with only human hand depth input. Su et al. (2021) propose multi-leap motion controllers and a Kalman filter-based adaptive fusion framework to achieve real-time control of an under-actuated bionic hand according to the bending angles of human fingers. After solving the retargeting problem, the recorded teleoperation data could be used as supervision data for imitation learning in Section 4.2.

#### 4.1.2. Human-Robot Correspondence Learning

In addition to the mapping methods above, some researchers directly utilize human demonstration data for reinforcement learning. Mandikal and Grauman (2020) first employ thermal images to learn affordance regions by human grasp demonstration and then take object-centric RGB, depth, affordance images/maps, and hand motor signals as inputs to a deep network with an Actor-Critic architecture to learn grasp policies. Garcia-Hernando et al. (2020) construct human hand depth images to robotic hands mapping dataset and adopt GAIL for end-to-end correspondence learning. The generator cascades a typical retarget module producing noisy poses and a residual RL agent to recorrect the action, which receives feedback from both the simulator and the discriminator. Moreover, the structure and control of soft robotic hands are much different from those of traditional robotic hands, which makes kinematic demonstration impossible. By leveraging object centric trajectories without hand-specific information as demonstrations, Gupta et al. (2016a) propose a Guided Policy Search (GPS) framework that could select feasible demonstrations to track and generalize to new initial conditions with nonlinear neural network policies.

### 4.2. Imitation Learning

For complex, sequential multifingered manipulation tasks with sparse, abstract rewards and high-dimensional, constrained state-action spaces, imitation learning is a promising way to achieve efficient learning. Note that in this section, demonstration recording and learning are executed in the same robotic hand.

#### 4.2.1. Hand Grasping Prediction

There are many analytic and data-driven methods tackling grasp points/boxes/cages estimation for simple two- or four-fingered grippers (Mahler et al., 2017; Fang et al., 2020), which is out of the scope of this article, as we focus on grasp affordance prediction for multifingered dexterous hands. GanHand (Corona et al., 2020) is a landmark model that can give a natural human grasp manner for each object in a cluttered environment by taking a single RGB image input of the scene. The detailed predictions for each object include its 3D model, all naturally possible hand grasp types defined in Feix et al. (2016) and the corresponding refined 51-DoF 3D hand models by minimizing a graspability loss. Generative Deep Dexterous Grasping in Clutter (DDGC) method (Lundell et al., 2021) has a similar structure as GANhand, but it adds depth channel and directly.

#### 4.2.2. Learning From Demonstration

**Figure 5:**
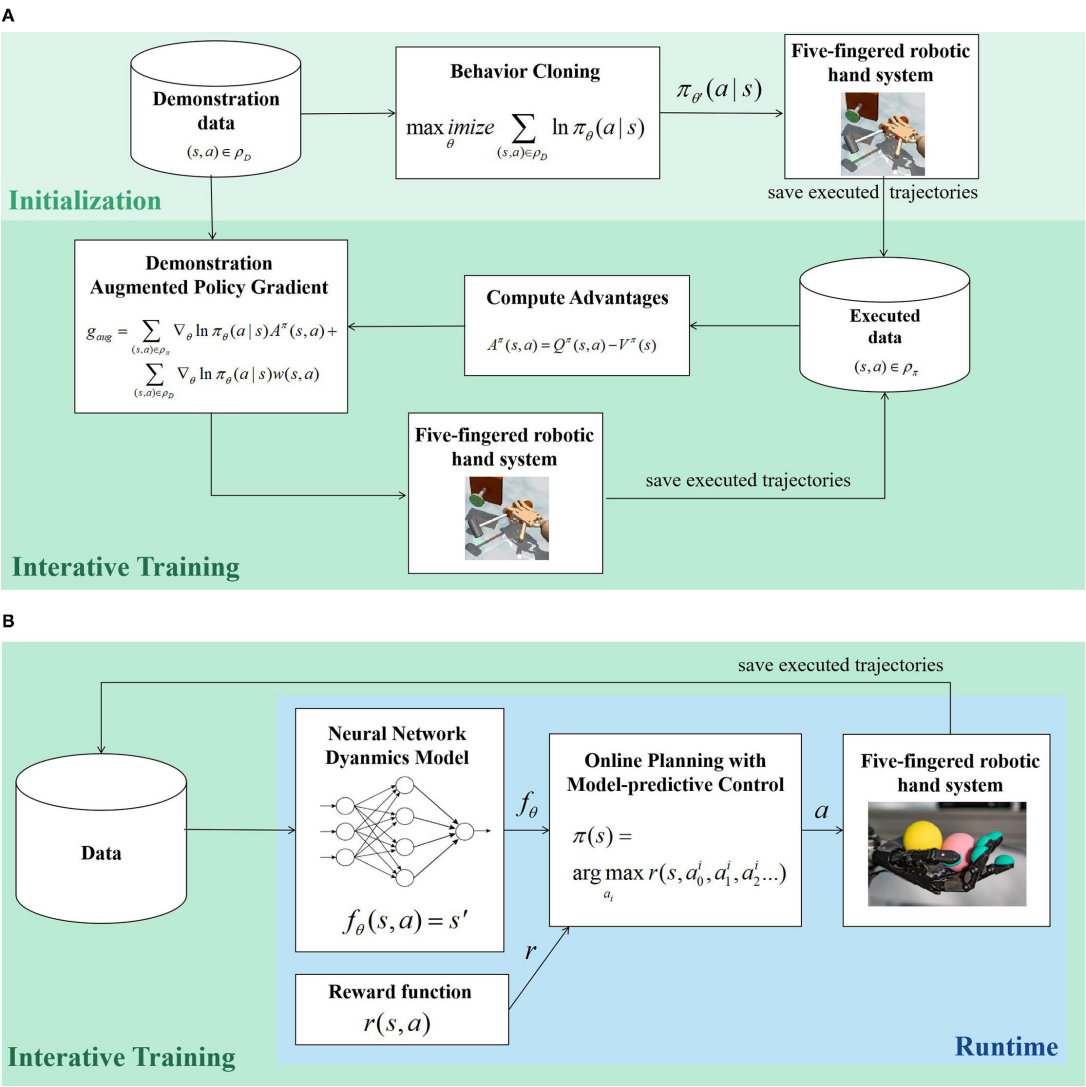
The brief diagrams of two learning-based five-fingered robotic hand manipulation methods. **(A)** A model-free, learning from demonstration method: Demo Augmented Policy Gradient (DAPG) (Rajeswaran et al., 2017), **(B)** A model based reinforcement learning method: Online planning with deep dynamics (PDDM) (Nagabandi et al., 2019).

To tackle the distributional shift of simple behavior cloning (BC), Demo Augmented Policy Gradient (DAPG) Rajeswaran et al. (2017), a model-free on policy method without reward shaping, first adopts BC for policy initialization and then integrates a gradually decreased weighted BC term into the original policy gradient term for further policy updates. A brief diagram of DAPG is given in Figure 5A. The sparse reward demonstration, learning, and verification of four tasks (object relocation, in-hand manipulation, door opening, tool us) are all conducted in a simulation environment with the Shadow Hand. Subsequent experiments in low-cost hand hardware, including Dynamixel claw and Allegro hand, also demonstrate the effectiveness of DAPG (Zhu et al., 2019). GAIL is also adopted for 29-DoF hand grasp learning (Antotsiou et al., 2019), in which the generator is trained with BC and Trust Region Policy Optimization (TRPO) sequentially; however, GAIL shows a low generalization for unseen initial conditions. Jeong et al. (2020) construct suboptimal experts by waypoint tracking controllers for 7-DoF bimanual robotic arms and learn primitives for 20-DoF robotic hands, and a general policy iteration method, Relative Entropy Q-Learning (REQ), is proposed to take advantage of the mixed data distribution of the suboptimal experts and current policies. As many data only have state information but no action labels, such as internet videos, Radosavovic et al. (2020) propose a state-only imitation learning method that interactively learns an inverse dynamics model and performs policy gradient. Osa et al. (2017) adopt initialized policy and create a dataset with contact information by human demonstration in simulation, and develop a hierarchical RL method for dexterous grasp with point cloud inputs, the upper-level policy selects the grasp types and grasp locations, and based on these, the lower-level policy generates the final grasp motions.

#### 4.2.3. Reward Shaping

Inverse reinforcement learning (IRL) is also a promising way to utilize demonstration data, however, there is rare work for its application in multi-fingered dexterous hands. Compromisingly, some researchers try to learn elaborate reward functions from demonstrations, which could work in combination with the standard RL method. Christen et al. (2019) propose a parameterizable multi-objective reward function including imitation reward and final state reward, in which position, angle, contact, and control terms are considered. By integrating with Deep Deterministic Policy Gradient (DDPG), policies are produced for five-fingered hand interaction tasks like a handshake, hand clap, and finger touch.

### 4.3. Reinforcement Learning

In this section, we investigate a) model-free and b) model-based reinforcement learning (RL) methods that learn from scratch for multifingered manipulation. Model-free methods are flexible, as they do not need to learn the model but require a large amount of trial and error. Model-based methods could use the model to guide exploration and policy search, which support re-planning and learning online and enjoy a high data efficiency.

#### 4.3.1. Model-Free Method

Different from Rajeswaran et al. (2017) that assume object and hand state values are known, Andrychowicz et al. (2019) use a hardware system with 16 tracking cameras to track hand fingertip location and 3 RGB cameras to estimate object pose based on multiview CNN. A distributed RL system based on Long Short-Term Memory (LSTM) and Proximal Policy Optimization (PPO) is proposed. By randomizing physical properties in different simulation environments, the learned discrete policies can easily be transferred to the physical Shadow Hand for in-hand manipulation tasks. For learning to be safe, Srinivasan et al. (2020) propose safety Q-functions for reinforcement learning (SQRL), in which safe policies could be learned during fine-tuning constrained by a learned safety critic obtained in the pre-training phase. Plappert et al. (2018) evaluate DDPG with and without Hindsight Experience Replay (HER) for in-hand manipulation and prove that DDPG integrating HER has a better performance with sparse rewards. To address reaching, grasping, and re-grasping in a unified way, Hu et al. (2020) deploy various quantified rewards and different initial states in training to experience failures that the robot could encounter, and a PPO and Proportional-Derivative (PD) controller are organized in a hierarchical way for dynamic grasping. Haarnoja et al. (2018) adopt Soft Actor-Critic (SAC), in which the actor aims to simultaneously maximize expected return and entropy, and the valve rotation task is estimated in an end-to-end DRL framework. Charlesworth and Montana (2020) introduce a trajectory optimization to generate sub-optimal demonstrations, which is combined with HER iteratively to solve precise dexterous manipulation tasks such as hand over and underarm catch. Rombokas et al. (2013) apply the policy search method, Policy Improvement with Path Integrals (PI2) to knob-turning task with a tendon-driven ACT hand and extend it to control in synergy-based reduced-dimensional space.

#### 4.3.2. Model-Based Method

Online planning with deep dynamics (PDDM) (Nagabandi et al., 2019) is a model-based method that iteratively trains a dynamic model with ensemble learning and selects actions with model predictive control (MPC). A brief diagram of PDDM is given in Figure 5B. For the Shadow Hand, by learning from scratch in simulation for 1-2 h or in real hardware for 2-4 h, the model could realize complex tasks such as handwriting and rotating two Baoding balls. By assuming a known accurate dynamics model, (Lowrey et al., 2019) propose a plan online and learn offline method (POLO), which utilizes local MPC to help accelerate and stabilize global value function learning and achieve direct and effective exploration.

### 4.4. Other Methods

In this section, we mainly review synergy-based methods and feedback-based methods. Other classical control methods will not be included.

#### 4.4.1. Synergy-Based Methods

Inspired by muscle synergies in neuroscience in Section 2.2.2, its modeling in robotic hand control with properties such as robustness, compliance, and energy efficiency is investigated by some researchers. Ficuciello et al. (2014) apply PCA to a reference set of 36 hand configurations and the first three principal components of each hand configuration are saved as the synergy components. Values of the synergy coefficients are computed with matrix manipulation and linear interpolation between the mean hand position configuration, the open hand, and the target hand configuration. A grasp quality index based on the force closure property is proposed as a feedback correction term (Ficuciello, 2019), which also acts as a reward function for Policy Improvement with Path Integrals (PI2)-based policy search of synergy coefficients (Ficuciello et al., 2016). Experiments are conducted with five-fingered under-actuated anthropomorphic hands, the DEXMART Hand and the SCHUNK S5FH, to achieve compliant grasps. However, this method requires the human operator to first place the object inside the opened hand in the except position. By further integrating human demonstration for initialization and neural network based object recognition, Ficuciello et al. (2019) proposed an arm-hand RL method for unknown objects in a synergy-based control framework. Geng et al. (2011) take the object position and pose information as input and adopt a three-layer MLP to output synergy coefficients for approaching and grasping respectively for the three-fingered Schunk robotic hand. In addition, soft adaptive synergies and synergy level impedance control methods are also implemented by researchers (Wimbock et al., 2011; Catalano et al., 2014). Katyara et al. (2021) expand synergies based methods to the whole precision grasp and dexterous manipulation process. First, Gaussian Mixture Model - Gaussian Mixture Regression (GMM-GMR) is applied to generate a synergistic trajectory that reproduces the taught postures, and kernelized movement primitives (KMP) are used to parameterize the subspaces of postural synergies for unknown conditions adaptation. For hand manipulation tasks with some fixed fingers, such as pen-knocking and spray, Higashi et al. (2020) construct a low dimensionality functionally divided manipulation synergy method and a synergy switching framework.

Moreover, Chai and Hayashibe (2020) try to analyze the emergence process of synergy control in RL methods, including Soft Actor-Critic (SAC) and Twin Delayed Deep Deterministic (TD3) methods. Three synergy-related metrics, Delta Surface Area (DSA), Final Surface Area (FSA), and Absolute Surface Area (ASA), are defined, and the results indicate that SAC has a better synergy level than TD3, which is reflected in higher energy efficiency.

#### 4.4.2. Feedback-Based Methods

Feedback-based methods could overcome insufficient and inaccurate perceptions in certain conditions and adjust control signals/policies online to recover from failures. Arruda et al. (2016) propose a multistage active vision grasping method for novel objects, grasp candidates are generated in a single view first and then grasp contact points and trajectory safety of reach-to-grasp are continually refined with switched gazes. Ganguly et al. (2020) use classical control formulations for closed-loop compliant grasping with only tactile feedback of the BioTac tactile sensors in the Shadow Hand. Additionally, for novel object grasping without seeing, Murali et al. (2020) first propose a localization method based on touch scanning and particle filtering and establish an initial grasp, and haptic features are learned with a conditional autoencoder, which is fed into a re-grasp model to refine the initial grasp. Zito et al. (2019) apply hypothesis-based belief planning for expected contacts even though the objects are non-convex and partially observable; if unexpected contact occurs, such information could be used to refine pose distribution and trigger re-planing.

Though we classify manipulation methods as the aforementioned concise categories. It is obvious that different methods could be flexibly combined to achieve better performance due to various task scenarios. For example, Li et al. (2019b) propose a hierarchical deep RL method for planning and manipulation separately. For rubik's cube playing task, the model based Iterative Deepening A* (IDA*) search algorithm is adopted to find the optimal move sequence, and the model free Hindsight Experience Replay (HER) method is taken as the operator to learn from sparse rewards. Human-robot hand poses retargeting is used to collect BC data in simulation for further imitation learning with DAPG (Rajeswaran et al., 2017). Some synergy-based methods also utilize RL methods such as policy search for synergy coefficients learning (Ficuciello et al., 2016).

## 5. Discussion and Open Issues

According to the previous review, the related discussions, open issues, and potential future directions of multifingered hands are discussed in this section.

### 5.1. Hardware Design and Simulation Modeling

**Hardware design:** As shown in Section 3, there are various dexterous hands designed for the disabled and robotic manipulation. However, there are still challenges in low cost, small size, arm-hand integration design, especially with the requirements of multimodal high-resolution sensory fusions (vision, force, touch, temperature, etc.) and object and task variations adaptation (size, shape, weight, material, and surface properties).

With the rapid development of manufacturing and processing technology, new materials and electronic, mechanical, electrophysiological components are emerging, which could provide new structures, actuators, and sensors for multifingered hand design. Moreover, the related biological findings (as shown in Section 2), such as the four types of tactile sensory receptors and the coupled, redundant musculoskeletal structure of human hands, could also further inspire the application of these new materials and components in robotic hands. Human hand-like or more general bioinspired robotic multifingered hands with high compliance and safety, low energy and cost, and various perception and manipulation abilities will be one promising direction.

From the structure aspect, artificial muscles such as contracting fibers with coiling-and-pulling functions and soft fingers with special actuators such as shape memory alloys and fluidic elastomer actuators (FEAs) could be further investigated for compliant, safe manipulation. Except for the manual grippers of humans, grippers of other creatures such as the spinal grippers of the snake body and the muscular hydrostat of the octopus arm (Langowski et al., 2020) could also inspired new structure of the dexterous hand. From the sensor aspect, soft skins with distributed tactile and temperature sensors in the finger tips as well as in the palm and all other regions that may contact objects could provide real-time, high-resolution interaction states between the hand and object and a cue of material properties of the object. Moreover, by taking into account the muscle and sensor structures of the human/animal hands and various evaluation metrics, handware and control co-optimization, which is an automatic learning of the optimal number and organization of the muscles and sensors as well as their control methods could also be meaningful for new hand prototypes compared to handcraft designs Chen et al. (2021). Some structure optimization works of musculoskeletal robots are highly related, such as the convex hull vertex selection-based structure redundancy reduction method (Zhong et al., 2020) and the structure transforming optimization method for constraint force field construction (Zhong et al., 2021).

**Simulation modeling:** Current simulation platforms such as Mujoco^6^, Gazebo^7^, and Webots^8^ provide authentic physical engines for demonstration data collection and training and verification of algorithms, which are efficient, low cost, and safe compared to direct learning in the hardware. However, two critical issues need further investigation: a) the modeling of new components and sensors, such as distributed tactile sensors, soft and deformable actuators, and materials, and b) decreasing the reality gap between simulation and real for seamless sim2real transferring and real-world applications.

### 5.2. Manipulation Control and Learning

The main challenges of multifingered hand manipulation control and learning include the high dimensional state and action spaces, frequently switched interaction, sparse hard-defined reward, and adaptation of various "internal" and "external" variations.

**Perception and cognition of state information:** Multimodal inputs such as RGB, depth, infrared images, cloud points and tactile senses of the object, hand and environments, and position, velocity, and acceleration of joints in robotic arm and hand are investigated by studies, some methods in Section 4 recognize the position, pose of object and hand (the demonstration hand) and the affordances explicitly, other methods encode the information in an end-to-end way directly for policy learning.

There is not a general framework for state information acquisition. A higher-level state represents abstract knowledge, while a low-level state includes more details. Thus, how to select proper types of sensors and design different levels of states for various tasks as inputs and supporting reward evaluation are worthy of further study. Meanwhile, related biological studies find various hand-centered visual modulation and visual-tactile fusion characteristics, such as the interaction between two visual pathways, object and non-object type neurons, as listed in Section 2.1. Building bio-inspired visual perception and cognition computational models for hand manipulation is also meaningful. In addition, EMG signals are also important inputs for prosthetic hand and human-robot skill transferring applications, in which, portable and sensitive EMG collected hardware design and elaborate signals and motor patterns recognition are also very important.

**Action synergy and advanced control:** Most learning-based methods take joints as independent components, which results in a high-dimensional action space that is difficult to learn (Rajeswaran et al., 2017; Nagabandi et al., 2019). These methods neglect the common knowledge in grasping taxonomy and the muscle synergies in the human hands, as given in Sections 2.2.3 and 2.3, which could be helpful for fast, compliant, robust, and energy efficient manipulation. Although there are some hand motor synergy studies, their modeling and task scenarios are limited (Section 4.4.1). Thus, learning the components and coefficients of synergies for the whole dynamic manipulation process remains challenging, and a new model structure and reward functions with synergy metrics may be needed. In addition, compared to common position control, with the new structure and actuators discussed above, flexible force control, impedance control and stiffness varying control for multifingered hands could introduce more compliance and robustness, and their fusion with RL will also be interesting. Some researchers have investigated the time-varying phasic and tonic muscle synergies for the musculoskeletal system, which is highly related and inspiring (Chen and Qiao, 2021).

**Model-based vs. model-free RL:** According to their pros and cons discussed in Section 4.3 and the related biological results of two discrete circuits for motor planing (Figure 3) given in Section 2.2. Design model-based and model-free fusion methods will also be an interesting research direction. Specifically, researchers need to model external clue-guided, goal-oriented model-based methods at a high level and habitual behavior- or instinct motivation-modulated model-free methods at a low level. Meanwhile, mechanisms for dynamically switching or fusing these two models should also be designed, and elements such as novelty and emotion could be considered. Some emotion-modulated robotic decision learning systems for reaching and navigation tasks have obtained promising results (Huang et al., 2018, 2021a,b).

**Learning from demonstration and imitation learning:** As continuous kinematics demonstration is exceedingly difficult for multifingered hands, how to collect and utilize the demonstration data is the key issue. If both state and action data are needed, a retargeting method is needed to transfer easily handled teachers, such as naked human hands or those with wearable apparatuses (gloves, makers, etc.), to the robotic hand, as reviewed in Section 4.1.1. During this process, both accurate pose recognition of hands with occlusion and appropriate mapping overcoming the structural discrepancies between different teachers and different robotic hands are challenging. Thus, on the one hand, designing a robust and adaptive retargeting method is necessary, in which a series of general mapping metrics may be critical. On the other hand, when suboptimal demonstration data are inevitable, which may also be generated much easier without elaborate design, learning from suboptimal or noisy data is worth further investigation. For under-actuated and soft robotic hands, retargeting is very difficult, perhaps only state data can be considered. IRL is a promising direction, which is rarely applied in multifingered hands, especially for multistage manipulation tasks in which it is difficult to define a value function. In addition, for portable and accurate body-arm-hand integrated movement recognition, other trackers and devices, such as the HTC Vive tracker and smart phone, should also be added (Qi et al., 2020).

Moreover, there are plenty of accumulated hand manipulation data in our daily lives, such as video data in YouTube, recorded data of monitoring systems in buildings, and vision systems of partner robots. Except for heterogeneous hand structures, this kind of “demonstration” always has different viewpoints, single modal (RGB and/or infrared) and includes complex multistage tasks; thus, how to utilize these data to supervise multifingered hand grasp and manipulation learning in its whole lifetime is a critical problem, and analyzing the mechanisms of mirror neurons may give some inspirations.

**Learning with adaptation**: Though the discussed methods in Section 4 could achieve learning-based manipulation to some extent, they lack the adaptation ability beyond common generalization. We believe the online correction, abstract task representation, transfer and evolution ability of manipulation skills are also important topics along with robotic applications in complex non-structured environments and human-cooperated situations. Specifically, for novel objects and environments with different arrangements or new obstacles, how to transfer the common manipulating characteristics and sub-models, adjust the actions in real time rather than reset from the beginning, achieve fast model updating by few-shot learning and avoid catastrophic forgetting are promising directions. Biological structures and mechanisms such as the hierarchical structure of motor control, and autonomy and amortized control ability of sub-regions in Section 2.2.3 may give some inspiration. We hypothesize that hierarchical RL and curriculum learning are potential frameworks (Zhou et al., 2021). Cloud robotics with shared memory and meta learning are also very related topics.

## 6. Conclusion

In this article, we investigate multifingered robotic manipulation from the aspects of biological results, structure evolution, and learning methods. First, biological and ethological studies of the various sensor-motor structures, pathways, mechanisms, and functions forcing the hand manipulation process (reach, grasp, manipulate, and release) are carefully investigated. Second, various multifingered dexterous hands are discussed from a new perspective: task scenario, actuation mechanism, and sensors for manipulation. Third, due to high the dimensionality state, and action spaces, frequently switched interaction modes and task generation demand for multifingered dexterous manipulation, learning-based manipulation methods consisting of LfO, learning by imitation, and RL are discussed. In addition, synergy- and feedback-based methods that are bioinspired and may benefit robust, efficient, and compliant control are also analyzed. Finally, considering the related biological studies and the shortcomings of current hardware and algorithms of multifingered dexterous hands, we discuss future research directions and open issues. We believe that biological structure, mechanism, and behaviors inspired multifingered dexterous hands and their learning and control methods will have prosperous outcomes in the future.

## Author Contributions

PW designed the direction, structure and content of the manuscript and wrote the abstract, introduction, and conclusion. YL investigated and wrote the related biological studies of visual sensing, the motor pathway of Section Biological Studies, and the manipulation learning method (Section Learning-Based Manipulation Methods). RL surveyed and wrote the biological studies of tactile sensing, the skeleton-muscle-tendon structure of Section Biological Studies, and their structural evolvements (Section Structural Evolvements). MT reviewed and wrote the biological studies of visual-tactile fusion and control mode of Section Biological Studies. ZL finished the discussion and open issues. HQ edited and revised the manuscript, and provided theoretical guidance. All the authors read and approved the submitted manuscript.

## Funding

This work is partly supported by the National Key Research and Development Plan of China (grant no. 2020AAA0108902), the National Natural Science Foundation of China (grant no. 62003059 and 61702516), the China Postdoctoral Science Foundation (grant no. 2020M673136), the Open Fund of Science and Technology on Thermal Energy and Power Laboratory, Wuhan 2nd Ship Design and Research Institute, Wuhan, P.R. China (grant no. TPL2020C02), the Strategic Priority Research Program of Chinese Academy of Science (grant no. XDB32050100), and the InnoHK.

## Conflict of Interest

The authors declare that the research was conducted in the absence of any commercial or financial relationships that could be construed as a potential conflict of interest.

## Publisher's Note

All claims expressed in this article are solely those of the authors and do not necessarily represent those of their affiliated organizations, or those of the publisher, the editors and the reviewers. Any product that may be evaluated in this article, or claim that may be made by its manufacturer, is not guaranteed or endorsed by the publisher.

## Footnotes

^1^ https://www.righthandrobotics.com/

^2^ https://www.ossur.com/en-us/prosthetics/arms/i-limb-quantum

^3^ https://www.ottobockus.com/prosthetics/upper-limb-prosthetics/solution-overview/bebionic-hand/

^4^ https://www.ottobockus.com/prosthetics/upper-limb-prosthetics/solution-overview/michelangelo-prosthetic-hand/

^5^ https://www.vincentsystems.de/evolution4

^6^ https://mujoco.org/

^7^ http://gazebosim.org/

^8^ https://www.cyberbotics.com/
